# Severe carvedilol toxicity without overdose – caution in cirrhosis

**DOI:** 10.1186/s40885-017-0083-z

**Published:** 2017-11-30

**Authors:** Satish Maharaj, Karan Seegobin, Julio Perez-Downes, Belinda Bajric, Simone Chang, Pramod Reddy

**Affiliations:** 1 Department of Internal Medicine, University of Florida College of Medicine, 4th Fl. LRC Building, 653 W 8th St, Jacksonville, Fl 32209 USA; 2 Jackson Memorial Hospital, Miami, USA

**Keywords:** Beta blocker, Carvedilol, Cirrhosis, Glucagon, Toxicity, Treatment

## Abstract

**Background:**

Carvedilol is used in the management of hypertension, ischemic heart disease, heart failure and most recently, portal hypertension. It has been associated with improved outcomes regarding variceal bleeding, hepatic decompensation and death when compared to propranolol and endoscopic band ligation. The main cause of portal hypertension is cirrhosis and therefore carvedilol is increasingly used in these patients. Due to its extensive hepatic metabolism, carvedilol is contraindicated in severe hepatic impairment. However, there are no dosage adjustments in the manufacturer’s labelling for mild to moderate hepatic impairment.

**Case presentation:**

We present a case of cardiogenic shock that occurred after carvedilol 25 mg orally was administered to a patient with cirrhosis. As there was no overdose, the diagnosis was based on clinical recognition of the toxidrome. The patient was successfully treated with glucagon 5 mg bolus followed by infusion.

**Conclusions:**

Patients with cirrhosis represent a special at-risk group for beta blocker toxicity. The typical threshold for carvedilol toxicity in overdose is 50 mg but in patients with cirrhosis this is not applicable. Nurses and physicians need to recognize the toxidrome early. Hospitals where carvedilol is used in patients with cirrhosis should have glucagon in formulary at doses to treat toxicity (bolus and infusion). Finally, dose adjustment and slow uptitration of carvedilol in cirrhosis is recommended.

## Background: Carvedilol use in patients with cirrhosis

Carvedilol is used in the management of hypertension, ischemic heart disease, heart failure and most recently, portal hypertension. Beta blockers decrease portal hypertension and are the mainstay of pharmacologic prophylaxis for gastroesophageal varices. The original beta blockers studied were nadolol and propranolol, but carvedilol recently emerged as an alternative [1]. There is also significant evidence that carvedilol is more potent with clinical benefits. Compared with propranolol, carvedilol was found to achieve greater reduction in hepatic venous pressure gradient [2] and work in non-responders to propranolol [3]. Carvedilol has been associated with improved outcomes regarding variceal bleeding, hepatic decompensation and death when compared to propranolol and endoscopic band ligation [3]. The main cause of portal hypertension is cirrhosis and therefore carvedilol is increasingly used in these patients.

## Case presentation

**Fig. 1:**
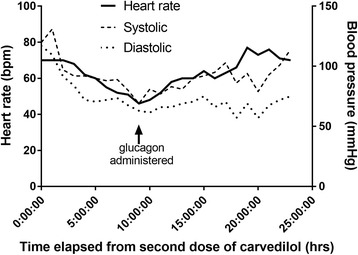
Graph showing heart rate and blood pressure after carvedilol 25 mg administered

A 56-year-old man with liver cirrhosis (secondary to hepatitis C) presented with hematemesis. The patient did not have diabetes, ischemic heart disease or other comorbidities. He did not regularly see a primary care physician and did not take any medications prior to admission. Laboratory investigation and imaging confirmed cirrhosis, with an alanine aminotransferase (ALT) of 101 IU/L, aspartate aminotransferase (AST) of 51 IU/L, albumin of 2.9 g/dL, total bilirubin of 1.4 mg/dL and international normalized ratio (INR) of 1.4. On imaging there was slight ascites and he did not have encephalopathy on examination, with Child-Pugh score of 7. An esophagogastroduodenoscopy confirmed bleeding esophageal varices, and band ligation was performed. Post intervention, the patient had an uneventful course and 7 days after presentation carvedilol 12.5 mg twice daily orally was started. Echocardiography on admission showed cardiac function was normal with a preserved ejection fraction. The other medications being administered were rifaximin and pantoprazole, and a final dose of ceftriaxone (1 g/day for 7 days) given only as prophylaxis. The first dose of carvedilol was administered after lunch and no reaction was noted acutely. At night the second dose was given. From this point on, the heart rate and blood pressure are charted in Fig. 1. At 2 hours after administration, the blood pressure declined from 120/78 mmHg to 97/61 mmHg but the heart rate was unchanged. Throughout the night the patient remained asymptomatic but the heart rate had started to decline. The patient denied any chest pain and the overnight team excluded myocardial ischemia with cardiac biomarkers and electrocardiography.

The following morning, 8 h after administration, the patient was hypotensive with a blood pressure of 80/45 mmHg, pulse of 51 beats/min and had a respiratory rate of 18 breaths/min. The patient was fully alert and oriented and expressed his wish to not have placement of any central venous access. A total of three liters intravenous crystalloid fluid boluses were administered but there was only a transient rise in blood pressure (Fig. 1, time 00 to 01 h). Electrocardiography and cardiac biomarkers again excluded myocardial ischemia. There was no leukocytosis, fever or tachypnea to suggest sepsis. Blood cultures demonstrated no growth. Serum biochemistry revealed normal electrolyte concentrations (K, Ca, Mg, Na) and no acute kidney injury.

On further investigation, the patient was hypothermic (33.7 °C) and hypoglycemic (serum glucose 40 g/dL) but the rest of the physical exam was unchanged. To note, the patient was not diabetic and had not received any insulin or other hypoglycemic medications. Laboratory investigations showed a stable hemoglobin and no signs of infection. Passive rewarming was started and intravenous dextrose and albumin given. Despite these measures repeat blood pressure was 69/42 and the patient began to become delirious. An electrocardiogram showed sinus bradycardia. A bolus of 5 mg intravenous glucagon was given. Within 5 min of administration the heart rate had increased to 65/min and blood pressure began to increase. This was followed by a glucagon infusion in 5% dextrose at 1 mg/h. As shown in Fig. 1, there was a steady response to treatment with clinical resolution. With a normal QT interval, the patient was pre-treated with 8 mg intravenous ondansetron to prevent vomiting associated with glucagon which did not occur. Repeat electrocardiogram during and after glucagon infusion showed normal sinus rhythm. The heart rate and blood pressure normalized and no further boluses of glucagon were required. There was concern as the nurse reported the patient had been anuric overnight. However, ultrasonography of the bladder showed that the patient was in urinary retention, which relieved with urethral catheterization. Vital signs on the following morning were blood pressure 126/56 mmHg, pulse 79 beats per minute and temperature 36.9 °C. There was no recurrence of hypoglycemia, hypothermia or urinary retention. The patient had no evidence of end organ sequelae and was discharged in stable condition.

## Discussion

### Pharmacokinetic considerations

Pharmacologically, carvedilol is a unique beta blocker. The formulation is a racemic mixture that forms S(−) and R(+) enantiomers which enable the drug to possess both non-selective β-adrenoreceptor antagonist and also α_1_-andrenoreceptor antagonist activity, respectively. Its vasodilating effect is theorized to contribute to its potency in reducing mortality and morbidity in the settings of ischemic heart disease and portal hypertension [4]. Compared to the pure β-adrenoreceptor antagonists, carvedilol’s α1-andrenoreceptor-antagonist activity may be both its strength and weakness. The vasodilating effect makes it more potent, but also presents a theoretical risk for additional hypotension in cases of overdose or supratherapeutic levels. In healthy individuals, after oral dosing, carvedilol is rapidly absorbed and undergoes extensive first-pass metabolism in the liver with a peak concentration 1 to 2 h after and subsequent hepatic metabolism [5].

### Safety and optimal dosing of carvedilol in patients with cirrhosis

As a therapeutic class the beta blockers have a good safety profile. Carvedilol has been widely used for decades, and at a dose of 25 mg daily, postmarketing surveillance has shown that it is generally well tolerated [6]. To the best of our knowledge, cardiogenic shock has not been reported with carvedilol use in therapeutic doses. Several clinical studies have investigated carvedilol in cirrhotic patients at varying doses. A summary of several studies is shown in Table 1 [2, 7–15]. Hypotensive events were noted both acutely and delayed and varied in incidence from 2.6% to 17.6%. These results imply that hemodynamic compromise can be a significant adverse effect of carvedilol at standard doses in cirrhosis.

**Table 1:** Hypotensive events in several studies investigating carvedilol in cirrhosis

Study (*n* = number of patients on carvedilol)	Daily dose of carvedilol (mg) studied	Follow up time(s)	Incidence of hypotension or bradycardia*
Hobolth et al. (21)	3.125–25 (mean 14 ± 7)	90 min; 92.7 ± 13.6 days	0
Stanley et al. (33)	6.25–12.5	30.7 months (7.9–47.1)	5 (15.2%)
Tripathi et al. (77)	12.5	26.2 ± 22.1 months	2 (2.6%)
De et al. (18)	12.5–25	90 min; 7 days	1 (5.6%)
Banares et al. (14)	25	60 min	0
Bruha et al. (36)	25	1 month	0
Forrest et al. (16)	25	60 min	0
Lin et al. (11)	25	90 min	0
Stanley et al. (17)	25	60 mins; 28 days	3 (17.6%)
Banares et al. (26)	6.25–50 (mean 31 ± 4)	11.1 ± 4.1 weeks	2 (7.7%)

Due to its extensive hepatic metabolism, carvedilol is contraindicated in severe hepatic impairment. However, there are no dosage adjustments in the manufacturer’s labelling for mild to moderate hepatic impairment. The pharmacokinetics of carvedilol are greatly altered in cirrhosis at both hepatic and systemic levels. Hepatic blood flow and drug extraction are reduced, leading to impaired metabolism and elimination of the drug. To add to this, carvedilol is a highly protein bound drug and hypoalbuminemia in cirrhosis affects the unbound serum concentrations.

Despite its widespread use in these patients, there is limited data on pharmacokinetics outside of healthy volunteers or patients with heart failure. Rasool et al. performed physiologically based pharmacokinetic modelling to simulate use in cirrhotic patients. They suggested that in these patients, to maintain drug exposure equivalent to 25 mg carvedilol in healthy individuals, the administered doses should be reduced to 12.5 mg, 6.25 mg, and 3.125 mg stratified by Child-Pugh class A, B, and C. Additionally, in liver cirrhosis the unbound systemic concentration of carvedilol increases much more in comparison to that of total systemic concentration of carvedilol [16].

Carvedilol decreases portal pressure after acute and long-term administration. Some authors have suggested that the clinical benefit of carvedilol, as reflected by reduction in HVPG, is directly proportional to dosage. In the studies examined (Table 1), investigators tended to initiate therapy at daily doses of either 6.25 mg (7–9,11) or 25 mg (2,10,12–15). Studies with a protocol that initially administered 25 mg daily dosing recorded a higher incidence of bradycardia and hypotension. From these studies as well as the case presented, we can suggest that a starting dose of 25 mg daily is too large a dose in patients with cirrhosis. We recommend initiating carvedilol at low dose, 3.125 mg twice daily, with close monitoring of heart rate and blood pressure that would require home monitoring and clinic visits. Slow uptitration at regular follow up visits can then be done as the patient tolerates.

### Carvedilol toxidrome and treatment with glucagon

Despite its widespread use, from our review of PubMed, ToxLine and International Pharmaceutical Abstracts, there are only two published case reports (English) of carvedilol toxicity and both of these were in the setting of overdose [17, 18]. The typical threshold for carvedilol toxicity in overdose is 50 mg [19] but in patients with cirrhosis this is not applicable. Especially in the absence of overdose, clinical recognition of the toxidrome is the key to early diagnosis leading to treatment. As in this case, the classic hallmarks of beta blocker toxidrome are hypotension and bradycardia, that can progress to cardiogenic shock and less frequently be accompanied by change in mental status, hypoglycemia or hypothermia. We were limited in this case because serum testing for carvedilol level was not available. However, the recognition of classic signs of beta blocker toxidrome and exclusion of alternative diagnoses allowed a clinical diagnosis. In practice, clinicians are unlikely to have access to carvedilol levels and prompt recognition of the toxidrome is essential to enable rapid treatment.

Carvedilol is a highly lipophilic drug and mental status should be monitored closely. In carvedilol toxicity, the signs of hypoglycemia are masked and a high index of suspicion is needed. With normal mental status and a stable airway, the focus of management should be on hemodynamic stabilization. We successfully used crystalloids and albumin followed by glucagon as a high dose intravenous bolus and infusion. Atropine has been recommended by some as first line treatment but has poor results in severe beta blocker toxicity [20]. In carvedilol toxicity, it is possible to use inotropes to maintain hemodynamic support. Bouchard et al. reported a case of overdose where the patient was responsive to glucagon boluses, 2-3 mg, but this was not followed by glucagon infusion [17]. Instead a dopamine infusion was started. Despite the dopamine infusion, however, the patient had occasional hypotension and bradycardia, and glucagon boluses were still used adjunctively with good response. If inotropes are used, it is suggested to select an agent based upon specific hemodynamic and cardiodynamic monitoring. There is no one catecholamine that is superior for cardiovascular drug toxicity but catecholamines such as isoproterenol and dobutamine, that possess predominant beta receptor activity and little alpha agonist activity may decrease peripheral resistance and worsen hypotension [20].

The treatment of beta blocker toxicity continues to be an area of active investigation. In beta blocker poisoning with bradycardia and hypotension, high-dose glucagon is considered the first-line antidote [21]. Glucagon has positive inotropic and chronotropic effects. Mechanistically, glucagon activates adenyl cyclase and exerts inotropic and chronotropic effects via a pathway not mediated by the adrenergic system. As Kerns [20] points out, this property makes glucagon particularly attractive as an antidote for beta blocker toxicity by providing cAMP necessary for myocardial cell performance in the face of beta blockade.

There are no studies on glucagon use in humans and current therapy is guided by animal studies and case reports. It would be unethical to undertake a randomized clinical trial investigating treatment of beta blocker toxicity. In the five studies of animal models of beta-blocker overdose in systematic review by Bailey, glucagon increased the heart rate (at least transiently) but appeared to have no effect on mean arterial pressure [22]. In this case we observed glucagon restoring the blood pressure (Fig. 1). When glucagon is used as an antidote, an “appropriate dose” should be administered. We agree that an appropriate dose represents a bolus of 5–10 mg followed by an infusion of 1–5 mg/h, titrated based on clinical response.

## Conclusions

Beta blocker toxicity is usually the result of overdose. Patients with cirrhosis represent a special at-risk group that can have toxicity at standard doses. Healthcare providers need to recognize the toxidrome early. In this case reversal was achieved with intravenous glucagon as evidenced by raised cardiac output and blood pressure. Hospitals where carvedilol is used in patients with cirrhosis should have glucagon in formulary at doses to treat toxicity (bolus and infusion).
